# Preventative practices and effects of the COVID-19 pandemic on caregivers of children with pediatric pulmonary hypertension

**DOI:** 10.1186/s12889-022-14651-2

**Published:** 2022-12-09

**Authors:** Erik J. Nelson, Ella Cook, Megan Pierce, Samara Nelson, Ashley Bangerter Seelos, Heather Stickle, Rebecca Brown, Michael Johansen

**Affiliations:** 1 Department of Public Health, Brigham Young University, 2148 LSB, Provo, UT 84660 USA; 2 Emma Eccles Jones College of Education & Human Services, Utah State University, Logan, UT USA; 3 Indiana University School of Medicine, Indianapolis, Indiana USA

**Keywords:** Pulmonary hypertension, COVID-19, Vaccination, Pediatric

## Abstract

**Background:**

Pulmonary hypertension (PH) is a serious and life-threatening disease characterized by elevated mean arterial pressure and pulmonary vascular resistance. COVID-19 may exacerbate PH, as evidenced by higher mortality rates among those with PH. The objective of this study was to understand the unique burdens that the COVID-19 pandemic has placed upon families of children living with PH.

**Methods:**

Participants were recruited online through the “Families of children with pulmonary hypertension” Facebook group and asked to complete a survey about their experiences during the COVID-19 pandemic.

**Results:**

A total of 139 parents/caregivers of children living with PH completed the online survey. Almost all (85.6%) of parents/caregivers had received the COVID-19 vaccine, though only 59.7% reported a willingness to vaccinate their child with PH against COVID-19. Over 75% of parents/caregivers felt that they practiced preventative measures (e.g., wearing a facemask, social distancing, and avoiding gatherings) more than those in the community where they live. They also reported several hardships related to caring for their child with PH during the pandemic such as financial duress, loss of work, and affording treatment costs.

**Conclusions:**

These findings indicate that parents/caregivers of children at higher risk for COVID-19 complications may be more willing to act on clinical recommendations themselves as proxy for protecting those at high risk. The economic, emotional and social impacts of COVID-19 are significantly greater for high-risk individuals.

## Background

Pulmonary hypertension (PH) is rare but a serious and potentially life-threatening disease characterized by an elevation in mean pulmonary artery pressure and pulmonary vascular resistance [1]. In severe cases, PH leads to right heart failure, clinical worsening, and death. PH is an important cause of morbidity and mortality in children, with an estimated annual incidence rate of approximately 3 cases per million children and is most commonly idiopathic or associated with congenital heart disease [2–9]. In children, untreated PH carries a grim prognosis, with subtypes such as idiopathic PH predicting a median survival of just 10 months [10]. PH has been reported in 23 to 37% of premature infants with chronic lung disease (CLD) in multiple retrospective studies, and carries a 2-year mortality rate as high as 48% [11–14]. In addition to substantial mortality risk, PH associated with chronic lung disease is associated with increased morbidity such as prolonged mechanical ventilation, need for supplemental oxygen, and increased hospital length of stay [11–18]. PH has primarily been characterized and studied among adults and therefore the development of treatment therapies has also targeted this population. Studies evaluating therapeutic dosing, side effects, and outcome for pediatric patients are lacking, however children are benefitting from therapies used to treat adults [19].

Since the onset of the coronavirus disease-19 (COVID-19) pandemic, millions of people have been infected with the severe acute respiratory syndrome coronavirus 2 (SARS-CoV-2) and millions have lost their lives as a result [20, 21]. Though COVID-19 incidence among those with PH appears to be similar to infection among the general population, the mortality rates for those with PH are significantly higher [22]. Children living with PH are thought to be particularly vulnerable to SAR-CoV-2 as it predominantly affects the respiratory tract, causing endothelial dysfunction, hypoxia and vasoconstriction. This further compromises the cardiopulmonary system, one that is already vulnerable due to the underlying pulmonary vascular disease [23]. A survey of 77 PH Comprehensive Care Centers estimated the incidence of COVID-19 in adults with PH to be 2.1 cases per 1000 people, though there is little data on the incidence of COVID-19 among children living with PH [24]. This is particularly concerning given the easy spread and communicability of SARS-CoV-2 [25]. Parents and caregivers of children living with PH have carried an even greater burden during this pandemic, as they not only administer detailed PH care plans, but also shoulder the responsibility of preventing SARS-CoV-2 infection in these children. In one retrospective cohort study of pediatric PH patients diagnosed with COVID-19, 35% required hospitalization due to clinical worsening or acute respiratory failure [26]. However, none of these patients required intubation. Though these patients showed excellent short-term recovery from COVID-19, the long-term consequences of long COVID on children living with PH [27] remain unknown, especially for this high-risk population of children living with PH [26].

The COVID-19 pandemic has negatively influenced infants and newborns with chronic conditions (such as PH) and their parents/caregivers, especially when clinics were temporarily closed during the pandemic leaving parents/caregivers with only telehealth/telemedicine visits to monitor their child’s condition(s) [23, 28]. Before the COVID-19 pandemic, approximately 10% of PH centers in the U.S. offered telemedicine visits, while 97% of PH centers offer virtual visits currently [23]. Since standard pediatric PH clinic visits include routine monitoring of vital signs, echocardiograms, stress tests, and laboratory evaluation, virtual appointments frequently do not meet the full needs of these patients. Thus, the crucial evaluation of the cardiopulmonary system and treatment therapy effectiveness were nonexistent, delayed or moved to irregular intervals as the healthcare system was inundated with changing guidelines, policies and recommendations to prevent the spread of COVID-19.

The goal of the current study was to understand the unique burdens that the COVID-19 pandemic has placed upon parents/caregivers of children living with PH. The focus of the study was to better understand the experiences of parents/caregivers accessing care, undertaking preventative practices against SARS-CoV-2 infection, beliefs towards COVID-19 vaccination, and disruptions such as education, doctor availability, prescription dispensing that impacted their ability to care for their child living with PH.

## Methods

### Facebook recruitment campaign

Participants were recruited online via Facebook™ invitations posted on the “Families of children with pulmonary hypertension” Facebook page. A random sample of group members (*n* = 934) also received a personal invitation via the internal Facebook messaging system (Facebook Messenger). To maximize study participation and to provide each group member an opportunity to participate, we posted the invitation on the Facebook group homepage 3 times over a 5-week period during December 2021 to January 2022. Participants who clicked on the invitation link were redirected to a secure study website and provided with study information, asked to convey consent to participate, and then immediately prompted to begin the survey using the Qualtrics™ survey platform. Eligible individuals who completed the survey were compensated for their participation and emailed a $20 gift card to Amazon.com®. The study protocol was approved by the Institutional Review Board at Brigham Young University (#IRB2021–371).

### Study participants

Parents and caregivers of children living with PH were surveyed about their personal experiences, as well as those of their child living with PH. Participants were English-speaking, aged 18 years or older, members of the “Families of children with pulmonary hypertension” Facebook group, and self-identified as parents, guardians or primary caregivers of a child living with PH. This Facebook group was selected because it is the largest known gathering place for parents of children living with PH (with approximately 1500 group members), many of whom routinely (daily) interact with others to learn more about existing PH therapies, to cope, and to share experiences of caring for a child with PH.

### Survey instrument

We developed a questionnaire containing questions intended to be answered by the parent of a child living with PH. The survey assessed participants’ knowledge, attitudes, perceptions, and burdens related to COVID-19 and caring for a child living with PH. Survey questions related to COVID-19 threat appraisal, vulnerability and anxiety were adapted from published studies for our study population [23, 29–31]. Sample survey items included “In your opinion, how effective are the following actions for keeping you safe from COVID-19”; “How often do you engage in the following practices to prevent COVID-19”; “Were you vaccinated against COVID-19”; “Will you vaccinate (or have you already vaccinated) your child who has PH against COVID-19”; and “How much do you trust the following sources to provide accurate COVID-19 information.” The majority of questions were assessed on a 5-point Likert scale ranging from 1 (*strongly disagree/never*) to 5 (*strongly agree/always*). In addition, we assessed specific familial hardships due to the COVID-19 pandemic including job loss (yes/no), loss of financial stability (yes/no), financial aid receipt to offset PH therapy costs (yes/no), and difficulty covering PH treatment costs (yes/no). Finally, we queried family sociodemographics including race (white/other), relationship to child living with PH (father, mother, guardian, primary caregiver), U.S. citizenship (yes/no), respondents’ educational attainment (high school, some college, associate’s degree, bachelor’s degree, graduate or professional degree), employment status (employed, homemaker, unemployed), household income (<$35,000, $35,000-74,999, >$75,000), health insurance status (yes/no). The average survey completion time was 19.5 minutes (range: 8 to 56 minutes).

### Statistical analysis

We calculated the frequency of actions undertaken by parents/caregivers and their child living with PH to prevent SARS-CoV-2 infection, reasons for not taking actions to prevent SARS-CoV-2 infection, attitudes and perceptions regarding COVID-19 vaccination for themselves and their child living with PH, sources from which parents/caregivers would seek COVID-19-related news and information, and financial, medical, and other impacts (i.e., job or insurance loss) imposed by the COVID-19 pandemic. We report the counts and frequencies (percentages) of each of the aforementioned actions. Statistical analysis was conducted in R version 4.1.0 [32].

## Results

Characteristics of the 139 parents/caregivers of children living with PH who responded to the online survey are presented in Table 1. The majority of respondents self-identified as White/Caucasian (82.0%), were mothers of the child living with PH (89.2%), had advanced educational attainment (60.5% had a college degree or higher), were U.S. residents (85.6%) and had health insurance (94.2%). Almost all parents/caregivers had received the COVID-19 vaccine (85.6%), though only 59.7% reported a willingness to vaccinate their child with PH against COVID-19. This was consistent with parent/caregivers’ attitude towards vaccinating other children in their home (57.6%). The primary reasons reported for not vaccinating their children against COVID-19 were the desire for more scientific evidence (39.5%), concerns about the safety of the vaccine (11.8%), and lack of trust in the expedited COVID-19 vaccine approval process (11.8%) (see Table 2).

**Table 1:** Characteristics of parents/caregivers of children living with pulmonary hypertension (*n* = 139)

Parent/Caregiver Characteristic	n (%)
**Age (years)**	
Mean (SD)	39.4 (8.73)
**Race/Ethnicity**	
White/Caucasian	114 (82.0%)
Other	25 (18.0%)
**Relationship to Child with PH**	
Father	10 (7.2%)
Guardian	3 (2.2%)
Mother	124 (89.2%)
Primary caregiver	2 (1.4%)
**Citizenship**	
U.S. Resident	119 (85.6%)
Outside U.S.	15 (10.8%)
Prefer not to say	5 (3.6%)
**Educational Attainment**	
High school graduate	9 (6.5%)
Some college, no degree	26 (18.7%)
Associate’s degree	18 (12.9%)
Bachelor’s degree	45 (32.4%)
Graduate or professional degree	39 (28.1%)
Prefer not to say	2 (1.4%)
**Employment Status**	
Employed	89 (64.0%)
Homemaker	34 (24.5%)
Unemployed	14 (10.1%)
Prefer not to say	2 (1.4%)
**Household Income**	
< $35,000	16 (11.5%)
$35,000 to $74,999	37 (26.6%)
> $75,000	76 (54.7%)
Prefer not to say	10 (7.2%)
**Health Insurance**	
Yes	131 (94.2%)
No	6 (4.3%)
Prefer not to say	2 (1.4%)

**Table 2:** COVID-19 vaccination uptake by parents/caregivers of children living with pulmonary hypertension (*n* = 139)

COVID-19 Vaccination	n (%)
**Are you vaccinated against COVID-19?**	
Yes	119 (85.6%)
No	16 (11.5%)
Prefer not to say	4 (2.9%)
**Would you vaccinate your child with PH against COVID-19?**	
Yes	83 (59.7%)
Maybe	11 (7.9%)
No	17 (12.2%)
Prefer not to say	5 (3.6%)
**Why would you not vaccinate your PH child against COVID-19? (*n*** **= 76)**	
Waiting for more scientific evidence	30 (39.5%)
COVID-19 Vaccine isn’t safe	9 (11.8%)
Do not trust the COVID-19 vaccine approval process	9 (11.8%)
Vaccines aren’t necessary	5 (6.6%)
Religious reasons	2 (2.6%)
Vaccines don’t work	2 (2.6%)

**Fig. 1:**
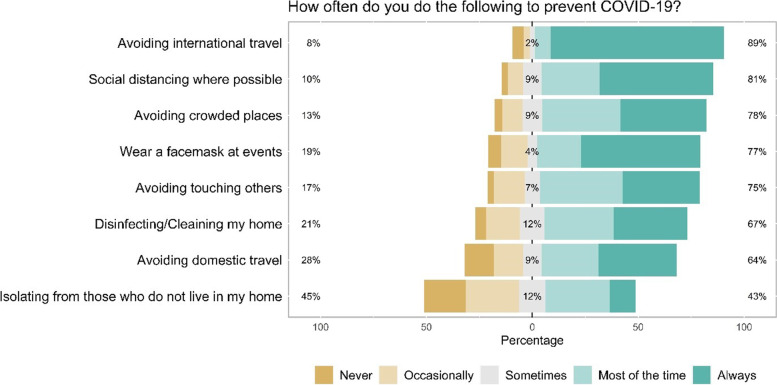
Practices of parents/caregivers of children living with pulmonary hypertension to prevent COVID-19 (*n* = 139)

**Fig. 2:**
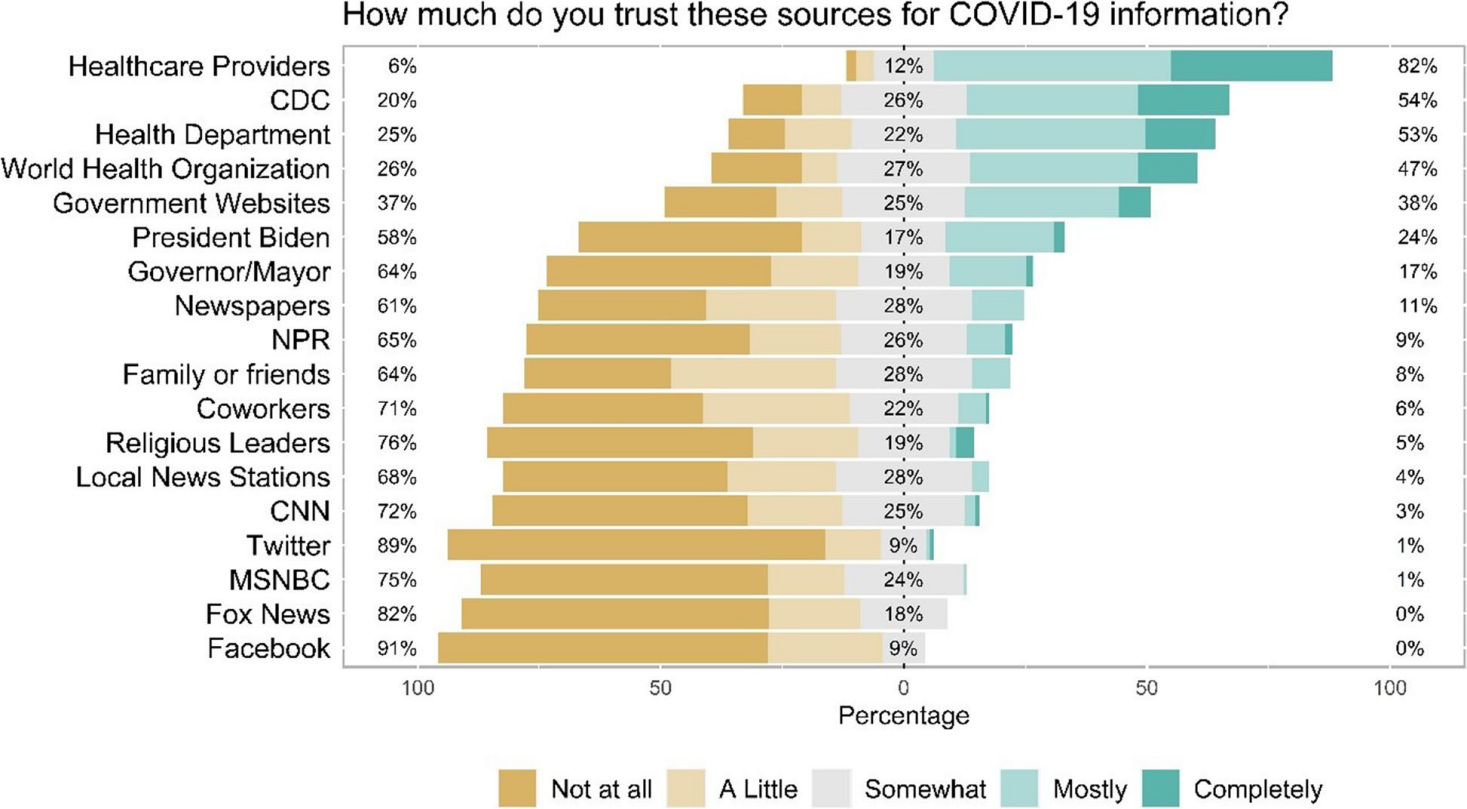
Trust of various sources for COVID-19 information reported by parents/caregivers of children living with pulmonary hypertension (*n* = 139)

Parents/caregivers engaged in a variety of preventative practices to prevent infection with SARS-CoV-2 (see Fig. 1). For example, parents/caregivers reported that they were engaging in the following behaviors always or most of the time: avoiding international travel (89.5%), practicing social distancing where possible (81.1%), avoiding crowded places (77.6%), wearing a facemask at social events (77.0%), and avoiding physical contact and touching others (75.3%). Over 75% of parents/caregivers felt that they practiced preventative measures such as wearing a facemask, social distancing, and avoiding gatherings and events more than those in the community did where they live. Parent/caregivers were also more likely to express trust in healthcare providers (84%) and official government sources [World Health Organization (58%), the Centers for Disease Control and Prevention (61%), and government-sponsored websites (50%)] than social media (0–6%), peers (11%), or local news stations (0–17%) to receive accurate information about the COVID-19 pandemic (see Fig. 2).

Parents/caregivers reported several financial hardships related to caring for their child with PH during the COVID-19 pandemic (see Table 3). For example, 41.0% of parents/caregivers reported financial duress and instability during the COVID-19 pandemic, 21.0% of parents/caregivers reported having lost their job during the COVID-19 pandemic, and 8.6% reported having difficultly covering the treatment costs for their child with PH. In addition, 71.9% of parents/caregivers thought their child’s education quality had decreased due to the COVID-19 pandemic, though the manner in which children with PH received their education (e.g., in-person schooling or virtual schooling) changed in less than half (41%) of the sample.

**Table 3:** Family hardships reported by parents/caregivers of children living with pulmonary hypertension (*n* = 139)

Family Hardships	n (%)
**Lost job during pandemic**	
Yes	29 (21.0%)
No	109 (78.9%)
Prefer not to say	1 (0.7%)
**Pandemic has affected financial stability**	
Yes	57 (41.0%)
No	79 (56.8%)
Prefer not to say	3 (2.2%)
**Received financial aid from a charitable organization**	
Yes	37 (26.6%)
No	100 (71.9%)
Prefer not to say	2 (1.4%)
**Difficulty covering treatment costs during pandemic**	
Yes	12 (8.6%)
No	124 (89.2%)
Prefer not to say	3 (2.2%)

## Discussion

We sought to understand the unique burdens of the COVID-19 pandemic and preventative practices against SARS-CoV-2 infection undertaken by parents/caregivers of children living with PH using a convenience sample from an online Facebook group. We found that 85.6% of parents/caregivers of children living with PH had received the COVID-19 vaccination, which is significantly higher than the 62.8% of vaccinated individuals in United States during the same period (January 1, 2022) [33]. This finding is consistent with vaccination rates among other high-risk populations such as adults ≥65 years (80.0%) [34], dialysis patients (74.1%), and pregnant women (66.8%) on January 1, 2022 [33]. Of note, this study concluded before the surge in COVID-19 cases and subsequent vaccinations that occurred in response to the highly contagious Omicron variant in early 2022 [33, 35]. We also found that 59.7% of parents/caregivers of children living with PH were willing to vaccinate their children against COVID-19. Importantly, 41.8% of children with PH in our study sample were less than 5 years old and were not yet eligible for the vaccine at the time of the study. It is worth noting that the COVID-19 vaccination has been shown to be safe and efficacious among children since the conclusion of our study [36, 37]. However, the willingness of parents to vaccinate their children with PH was much higher than that reported vaccination rate among children in the United States (28.1%) at the time of the survey [38, 39], yet far below the parental vaccine intention attitudes (86.7% of parents reported they would definitely or probably vaccinate their children) that have been suggested elsewhere [40]. These findings indicate that parents/caregivers of children at higher risk may be more willing to act on clinical recommendations themselves as proxy for protecting their children than parents of children who do not have elevated risk from SARS-CoV-2 infection despite initial straw polls of parental attitudes towards COVID-19 vaccination.

Parents/caregivers of children living with PH were also engaged in several recommended prevention practices and at higher rates than most adults in the U.S. [41, 42]. In lieu of vaccination, these practices have been the most effective evidence-based practices to reduce the transmission of COVID-19 [43, 44]. Importantly, parents/caregivers were surveyed approximately 21 months into the pandemic, yet they reported maintaining these practices despite the fatigue, letdown and abandonment of these practices by many adults. This may be partially explained by the perceived elevated risk and/or fear of serious complications if the child with PH were to become infected with SARS-CoV-2. The longevity of practicing these behaviors has been directly linked to depression and poorer mental health outcomes [45, 46], which may be exacerbated by the financial, temporal, and emotional tolls of caring for a child with PH as has been observed in other studies [47–49].

The large majority (71.9%) of parents/caregivers of children living with PH reported that the quality of their child’s education has decreased due to the COVID-19 pandemic. This educational deficit is not unique to children living with PH, though this may further isolate children with PH as they are at greater risk of serious complications from SARS-CoV-2 infection. For example, since many children with PH are not able to participate in common childhood activities (i.e., running, many contact sports, riding a bike) and now must maintain social distance, they may experience disproportionate social development and educational setbacks [50]. Apart from educational impacts, parents/caregivers also reported hardships such as job loss, difficulty covering PH medications and therapies, and overall financial instability. Again, these impacts of the COVID-19 pandemic are not unique to families of children with PH, but they do highlight the extra burden shouldered by caring for a child with a chronic health condition [28, 51].

This study has some limitations. First, parents/caregivers were recruited from an online Facebook group and are a convenience sample (i.e., not representative) of all parents/caregivers of children living with PH. However, this is also a strength as this is the largest group worldwide of individuals with children living with PH. Second, survey responses were self-reported by parents/caregivers over the Internet and may be subject to under or overreporting. However, other Internet-based studies have shown increased self-disclosure and reporting with online surveys, which may reduce potential response biases (e.g., interviewer bias or social desirability) [52, 53]. Finally, the cross-sectional study design does not permit causal inference.

This study enhances our understanding of how parents/caregivers of children living with PH have responded to the COVID-19 pandemic, including preventative practices and vaccination uptake. The results demonstrate how parents/caregivers may respond differently to COVID-19 recommendations and practices in order to protect high-risk individuals under their care and supervision. Learning about the experience of these individuals living through the COVID-19 crisis can help empower public health and healthcare professionals to continue to protect the health of this population.

## Data Availability

We are not allowed to post the data to a repository (even de-identified) due to HIPAA concerns (potential to identify subjects). Data and materials available upon request to the corresponding author.
